# Self-reflection Orients Visual Attention Downward

**DOI:** 10.3389/fpsyg.2017.01506

**Published:** 2017-09-05

**Authors:** Yi Liu, Yu Tong, Hong Li

**Affiliations:** ^1^College of Psychology and Sociology, Shenzhen University Shenzhen, China; ^2^China Center for Special Economic Zone Research, Shenzhen University Shenzhen, China; ^3^School of Psychological and Cognitive Sciences, Peking University Beijing, China

**Keywords:** self, attention orientation, above-average, self-enhancement, social context

## Abstract

Previous research has demonstrated abstract concepts associated with spatial location (e.g., God in the Heavens) could direct visual attention upward or downward, because thinking about the abstract concepts activates the corresponding vertical perceptual symbols. For self-concept, there are similar metaphors (e.g., “I am above others”). However, whether thinking about the self can induce visual attention orientation is still unknown. Therefore, the current study tested whether self-reflection can direct visual attention. Individuals often display the tendency of self-enhancement in social comparison, which reminds the individual of the higher position one possesses relative to others within the social environment. As the individual is the agent of the attention orientation, and high status tends to make an individual look down upon others to obtain a sense of pride, it was hypothesized that thinking about the self would lead to a downward attention orientation. Using reflection of personality traits and a target discrimination task, Study 1 found that, after self-reflection, visual attention was directed downward. Similar effects were also found after friend-reflection, with the level of downward attention being correlated with the likability rating scores of the friend. Thus, in Study 2, a disliked other was used as a control and the positive self-view was measured with above-average judgment task. We found downward attention orientation after self-reflection, but not after reflection upon the disliked other. Moreover, the attentional bias after self-reflection was correlated with above-average self-view. The current findings provide the first evidence that thinking about the self could direct visual-spatial attention downward, and suggest that this effect is probably derived from a positive self-view within the social context.

## Introduction

“Look down and see the beggars at your feet, look down and show some mercy if you can.”

- - - - - - -From *Les Miserables*.

Nobles in the upper class look down with pride to see others at the bottom of the heap, while those of the underclass, look up to dignitaries, and show their respect. “Up” and “down” are not only used for concrete spatial location, but also as metaphors for abstract concepts, such as attitudes toward others (e.g., “look up to the leader” and “look down upon the beggar”). These kinds of metaphors have also been used to describe the higher position one possesses relative to others within the social hierarchy. For example, “I am at the top of the class” or “I am above average people.” The belief that we are above average is a robust cognitive bias that helps to maintain self-esteem (Alicke, 1985; Taylor and Brown, 1988; Chambers and Windschitl, 2004; Beer and Hughes, 2010). In the current study, we focused on the spatial metaphor of the superiority of the self, and demonstrated its influence on visual attention.

Psychological researchers have been interested in the influence of abstract concepts on visual attention. These abstract concepts include ones associated with vertical spatial information, such as God and devil. Individuals responded faster to target stimuli presented at compatible locations, such as God is up and devil is down (Meier et al., 2007; Chasteen et al., 2010). Meier and Robinson (2004) also demonstrated that positive/negative words (e.g., hero/liar) were evaluated faster when they appeared at the up/down position of the screen. Using pictorial stimuli, Schubert (2005) showed that power was aligned to a vertical schema, in which a powerful agent (e.g., master) is on top of a powerless one (e.g., servant). These findings demonstrate that abstract concepts with implicit spatial information could trigger automatic visuospatial attention orientation toward locations compatible with their meanings. In the social context, to maintain self-esteem, people also tend to show cognitive bias that place themselves as better or “above” average people (Alicke, 1985; Taylor and Brown, 1988; Chambers and Windschitl, 2004; Beer and Hughes, 2010). This positive bias of the self suggests an association between the self-concept and an above-average position within the social context. However, whether thinking about self could show similar attention orientation effects is still unknown. Previous research has demonstrated that self-reflection on personality traits resulted in self-bias in memory, which is called “self-reference effect.” That is, compared with other-related trait adjectives, self-related traits were better remembered, suggesting that self functions as a superordinate schema deeply involved in memory (Rogers et al., 1977; Klein and Kihlstrom, 1986). Investigating whether self-reflection could result in self-bias on visuospatial attention is crucial for understanding self-reflection. Attention orientation effect provides a good way to investigate this issue.

A theoretical account for the attention orientation effects of abstract concepts is the Perceptual Symbol Systems (PSS) theory, introduced by Barsalou (1999). The PSS theory proposes that our perceptual systems capture sensorimotor information during processing of a stimulus (e.g., the clouds are up in the sky). Then, during the conceptualization process (e.g., thinking about clouds in mind), the same perceptual systems are activated and the sensorimotor state is reenacted (e.g., looking up). For abstract concepts without a concrete physical basis (e.g., God), we often use sensory-based metaphors to describe them. Therefore, cognitive processes rely on these perceptual metaphors (Lakoff and Johnson, 1999) and the representational processes that are embodied in nature (Boroditsky and Ramscar, 2002). Using the study by Meier et al. (2007) as an example, God is closely tied to a vertical representation processes because we cannot directly perceive God, thus the conceptualization strongly relies on a perceptual metaphor, such as God is the “most high” and resides in the “high Heavens.” The activation of spatial cues when thinking about God in this study increased the speed of responses to targets at the upper position. While for the self-concept, the “above average” cognitive bias for the self and look “down” upon others during social comparison reminds us of the higher position in which we exist relative to others within the social group. Thus, this raises the questions of whether thinking about the “self” directs visuospatial attention, and if so, in which direction (up or down) attention is oriented.

In previous studies, visual attention was guided by the position of external objects or concepts, using the self as the frame of reference. For example, God is located above the earth on which the self resides, so one would look up for God. Since the “above average” bias of the self uses others as the frame of reference, and the existence of others below us builds our sense of pride in the social environment, the higher position of the self would lead us to look down for others to maintain the superiority of the self. From the view of clinical psychology, when patients look up to the analyst (e.g., when lying down on the coach), the posture increases their feeling of smallness. By contrast, when the patients’ posture allowed them to look down on others, the superiority of the self will be established (Steiner, 2006). Thus, we hypothesized that thinking about the self would direct visual attention downward.

The aim of the current study was to test whether thinking about the self could direct visual attention, and if so, in which direction (up or down) would attention orient. We adopted the target discrimination task (Estes et al., 2008) after self- or other-reflection on personality traits to investigate this issue. In Study 1, we used the self and a friend as target persons to show the effects of self-reflection and other-reflection on visual attention. The self-specific attention orientation effect was expected. Because our participants were all from an Eastern culture, in which individuals tend to regard their friend as part of the self (Markus and Kitayama, 1991), in Study 2, we used a disliked other as a control to replicate the attention orientation effect after self-reflection. Moreover, in Study 2, we also measured the subjective status of participants using the above-average paradigm (Beer and Hughes, 2010) to confirm that the attention orientation after self-reflection was derived from a positive self-view.

## Study 1

### Methods

#### Participants

Forty-two adults (19 males, mean age = 21.12 years, standard deviation *SD* = 1.89 years) participated in this study for monetary compensation. All participants were right-handed and had normal or corrected-to-normal vision. Written informed consent was obtained prior to participation. This study was approved by the ethics committee of Shenzhen University. In previous studies on attention orientation, the η^2^ were approximately 0.2 in 2 × 2 designs (Meier et al., 2007; Estes et al., 2008; Gozli et al., 2013). An *a priori* power analysis using G^∗^Power (Faul et al., 2009) revealed that assuming an η^2^ = 0.2, 34 participants were adequate to detect a medium- to large-sized interaction, with an *α* of 0.05, and power of 0.80.

#### Stimuli and Procedure

##### Attention Orientation Task

**FIGURE 1:**
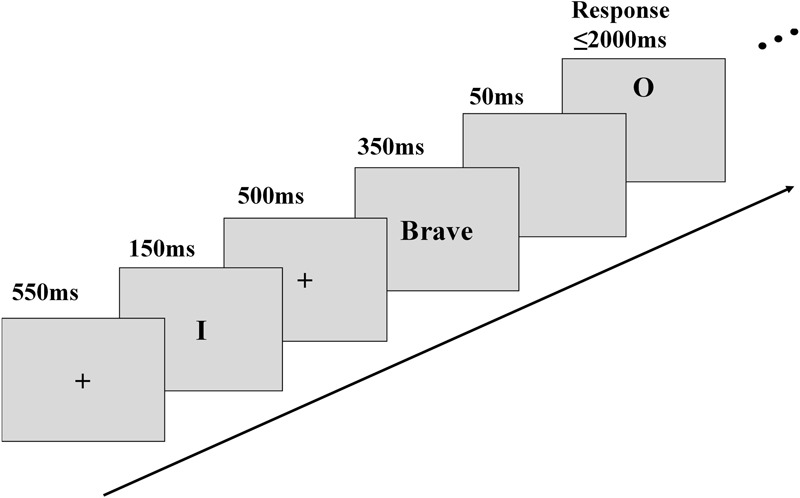
Illustration of experimental procedure of the attention orientation task.

We selected 64 personality trait adjectives (32 positive and 32 negative) from an established personality trait adjective pool (Liu, 1990). These 64 words were classified into two blocks, one positive block with 32 positive adjectives, and one negative block with 32 negative adjectives. Each block was presented twice, once for self and once for a gender-matched friend. The “friend” condition existed as a control to clarify whether the attention orientation effect of trait reflection was self-specific. The four blocks (self-positive, self-negative, friend-positive, friend-negative) were presented in a random order, and with a 10 s interval between each two blocks. On each trial, a 550 ms fixation was followed by the word of “I” or “Friend” presented at the center of the screen as a person cue for 150 ms. Then, a trait adjective describing the person was presented for 350 ms in the center of the screen subtending 1° of the visual angle. After a 50 ms delay, a target letter “X/O” appeared at the upper or lower position of the screen relative to the fixation, 2.3° vertically from the center of the display. The target letter was presented for 2000 ms as the longest duration waiting for responses (**Figure 1**). Participants were asked to think about the trait descriptions for the cued person and judge the target was X or O by pressing one of the two buttons using their right hand when the letter appeared. Both accuracy and speed were instructed. The target (X or O) and its position were balanced within subjects and the button press was balanced across subjects.

In this paradigm, attention orientation speeds up the responses to targets at positions congruent with the spatial association of the concepts and slows down the responses to targets at incongruent positions (Meier and Robinson, 2004; Meier et al., 2007; Estes et al., 2008). Thus, in current study, the expected downward attention orientation should be defined as the differential RTs to higher targets and lower targets after self-reflection.

##### Questionnaires

According to our hypothesis, the attention orientation effect was rooted in the psychological status one holds of the self within the social context. We therefore measured the socioeconomic status (SES) of participants, as an index of subjective social status, and measured self-construal as an index of the sensitivity to social information. SES were measured using the subjective SES scale (Adler et al., 2000) with the drawing of a ladder of 10 rungs. People at the higher position of the ladder have more money, higher level of education, and better jobs than people at the lower position of the ladder. Participants were asked to indicate the position they feel they stand on the ladder. Self-construals were measured using the Self-Construal Scale (SCS, Singelis, 1994), which consists of 24-items that assess independent and interdependent self-construals, on a 7-point Likert scale (1 = *strongly disagree*, 7 = *strongly agree*). In addition, we also asked participants to rate to what extent they were familiar with, and similar to the friend, as well as how much they liked this friend, on 8-point scales (1 = *not at all*, 8 = *very much*).

### Results

Inaccurate trials in the X/O discrimination task were removed from data analysis, and the reaction times (RTs) were log-transformed to normalize distribution (Ratcliff, 1993). Trials that were 2.5 SDs below or above the mean RTs for each subject were also removed from data analysis (similar to Meier and Robinson, 2004). The mean accuracy was high (95.94%) and the average removal rate was 6.19%.

**FIGURE 2:**
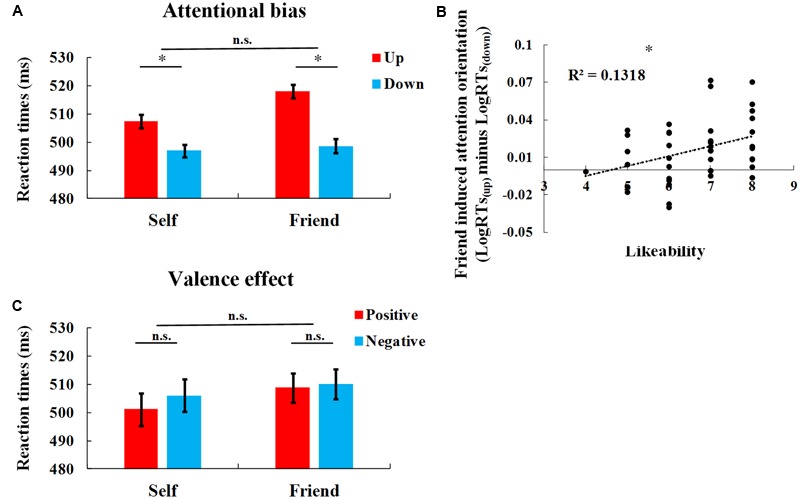
Results of study 1. **(A)** RTs to targets in different conditions. The RTs are reported in raw millisecond values to facilitate comprehension. Error bars represent within-subject standard error of the mean. **(B)** Correlation between downward attention orientation effect (lgRT_up_-lgRT_down_) after friend-reflection and the likeability of the friend. **(C)** RTs to targets after positive and negation reflection. The RTs are reported in raw millisecond values to facilitate comprehension. Error bars represent within-subject standard error of the mean. ^∗^*p* < 0.05.

The mean RTs and SDs are reported in **Table 1** and the log-transformed RTs were subjected to a 2 (Person: self vs. friend) × 2 (Valence: positive vs. negative) × 2 (Position: up vs. down) repeated measures analysis of variance (ANOVA). The main effect of Position was significant [*F*(1,41) = 19.265, *p* < 0.001, η^2^ = 0.320], with faster responses to the targets at the lower position than those at the upper position (*p* < 0.001). This effect of Position was significant for both self (*t*(41) = 2.440, *p* = 0.019, *d* = 0.379, 95% confidence interval of the difference (CI) = [-0.0243, -0.0090]), and friend (*t*(41) = 4.393, *p* < 0.001, *d* = 0.678, 95% CI = [-0.0170, -0.0016]; **Figure 2A**). The differential RTs to targets at up vs. down positions for individual subjects are demonstrated in **Supplementary Figure S1**. However, other main effects or interactions were not significant [Person: *F*(1,41) = 0.201, *p* = 0.656, η^2^ = 0.005; Valence: *F*(1,41) = 0.167, *p* = 0.685, η^2^ = 0.004]; Person × Valence: *F*(1,41) = 0.092, *p* = 0.763, η^2^ = 0.002; Person × Position: *F*(1,41) = 1.972, *p* = 0.168, η^2^ = 0.046, **Figure 1**; Position × Valence: *F*(1,41) = 1.492, *p* = 0.229, η^2^ = 0.035; Person × Valence × Position: *F*(1,41) = 2.929, *p* = 0.095, η^2^ = 0.067). These results suggest downward attention orientation after both self- and friend-reflection. The attention bias of self was consistent with our hypothesis, however, the similar effect of the friend was unexpected. One possibility of the similar effect of friend-reflection was the “friend” was regarded as part of the self, thus similar attention orientation to that seen in the “self” condition was observed. We noticed that friend-reflection induced downward attention orientation (lgRT_up_-lgRT_down_) was positively correlated with the likeability scores of the friend [*r*(41) = 3.63, *p* = 0.020; **Figure 2B**], that is, the more an individual liked the friend, the stronger attention orientation effect was induced by friend-reflection. Additionally, we checked the self-construals of the participants. The self-construal scores were higher in interdependent than in independent subscales (interdependent: 61.45 ± 7.34, independent: 54.93 ± 7.18, *t*(41) = 5.471, *p* < 0.001, *d* = 0.844, 95% CI = [4.116, 8.932]). It is well known that interdependent individuals tend to regard a friend or close other as a part of the self (Markus and Kitayama, 1991). Thus, these results suggest that the attention orientation effect observed following friend-reflection relies on the attitude of the individual (i.e., likeability) toward the friend, and possibly, this attitude is rooted in the individual’s self-construal. The possible confounding of a general attention facilitation to lower positions is more thoroughly addressed in Study 2.

**Table 1:** Mean RTs and SDs in the attention orientation task.

Mean (*SD*)		Self-positive	Self-negative	Friend/disliked	Friend/disliked
(ms)				positive	negative
Study 1	Up	505.42 (88.32)	509.70 (85.95)	513.63 (97.88)	524.40 (110.81)
	Down	492.41 (76.59)	500.25 (92.03)	505.96 (116.97)	496.64 (93.74)
Study 2	Up	517.45 (81.83)	552.93 (92.88)	521.62 (92.20)	518.73 (68.97)
	Down	502.96 (76.38)	539.28 (87.73)	522.93 (116.79)	521.27 (73.07)

Other ratings or questionnaires were not correlated with self- or friend-reflection induced attentional orientation (*p*s > 0.1).

Identical analyses were conducted on accuracy data. The 2 × 2 × 2 ANOVA did not show any significant main effects or interactions (*p*s > 0.1). The accuracy results suggest that there was no speed-accuracy trade-off for the faster responses to targets appearing at the lower position.

### Discussion

As expected, we found self-reflection resulted in faster responses to targets that appeared at a lower position compared with responses to targets at higher position, suggesting a downward attention bias after self-reflection. However, a similar effect was also found after friend-reflection. There were two possibilities to explain these results. First, it is possible that the attention orientation was induced by general trait evaluation or just a facilitation effect on the targets appearing at the lower position. A second possibility is that the attention orientation was specifically related to self-concept. In Eastern cultures, friends are often considered as part of the self (Markus and Kitayama, 1991), thus resulting in the similar effect observed between the reflection on self and the friend. Here, we prefer the latter possibility, as there was evidence showing that self-enhancement could extend to others who are construed as part of self. For example, Gardner et al. (2002) manipulated self-construals and found that, when participants were primed to hold an expanded sense of self that included close others, they expected better performance for their friend as well as for themselves. This means that, if our participants regarded the friend as part of the self, they would also hold a positive view of their friend. Thus, it is reasonable to show similar attention orientation after friend-reflection. Our participants were more interdependent than independent in self-construals, and evidence that individuals who liked their friend more showed stronger attentional bias, supports this possibility. To explore this further, in Study 2 we replaced the friend with a disliked other to test whether the downward attention bias is specific to the self, or instead, general to all persons.

Additionally, we did not find correlations between attention orientation effects and the social status measured with the SES scale. The SES scale contains only one question (indicating a position they stand on the ladder) integrating information regarding money, education, and jobs, but with no evaluation of personality traits. Since the positive bias of self typically refers to an above-average view of personality traits (Beer and Hughes, 2010), we inferred that the SES scale may not be a sensitive index of positive self-view within the social context. Therefore, in Study 2, we adopted the above-average paradigm to measure the subjective social status considering personality traits, and correlated the above-average self-view with the attention orientation effect following self-reflection.

## Study 2

In Study 1, although we observed the expected downward attention orientation after self-reflection, the similar results of the friend condition confounded the effects with the possibility that the attention orientation was a general facilitation effect on targets at lower positions. To rule out this confound, we conducted Study 2. In this study, a disliked other was compared with the self to test whether the downward attention orientation is general to all persons or self-specific. Moreover, theoretically, we inferred that the downward attention orientation was due to the automatic tendency of self-enhancement during social comparison. Therefore, in Study 2, we also measured self-enhancement effects, using the above-average paradigm, to test whether downward attention orientation after self-reflection were correlated with above-average self-view.

### Methods

#### Participants

Forty-two adults participated in this study for monetary compensation. Gender and age were matched to the participants in Study 1. One participant was deleted because of low accuracy (6.25%), resulting in 41 total participants (19 males, mean age = 21.24 years, *SD* = 2.23 years) included in data analysis. One participant was left-handed, and all others were right-handed, and all had normal or corrected-to-normal vision. Written informed consent was obtained prior to participation.

#### Stimuli and Procedure

##### Attention Orientation Task

The stimuli and procedure were identical to Study 1, except that a “disliked” other was used as a control. The disliked other was defined as a gender-matched person, known to the participant in daily life, but whom the participant did not like. For example, the participant may disagree with his or her ideas or behaviors. The cue word presented to indicate the disliked person was “he/she.”

##### Above-Average Measurement

To test whether downward attention bias is related to the psychological status or position one possesses within their specific social environment, we also measured the subjective status of participants, using the above-average paradigm (Beer and Hughes, 2010). Forty-eight positive trait adjectives were selected from the personality trait adjective pool (Liu, 1990). The participants were asked to judge, in relation to the average students in their university, to what extent they were above or below the average on each personality trait. An 8-point scale was used for the judgments, from *much lower* to *much higher*. Each adjective was presented on the screen until participants made their judgments. The inter-trial fixation was presented for 500 ms.

##### Questionnaires

The questionnaires (i.e., SES, SCS) used in Study 1 were also used in Study 2.

### Results

#### Attention Orientation Task

Similar to Study 1, inaccurate trials in the target discrimination task were removed from data analysis, and the RTs were log-transformed to normalize distribution (Ratcliff, 1993). Trials that were 2.5 SDs below or above the mean RTs for each subject were also removed from analysis. The mean accuracy was high (95.46%) and the average removal rate was 6.63%.

**FIGURE 3:**
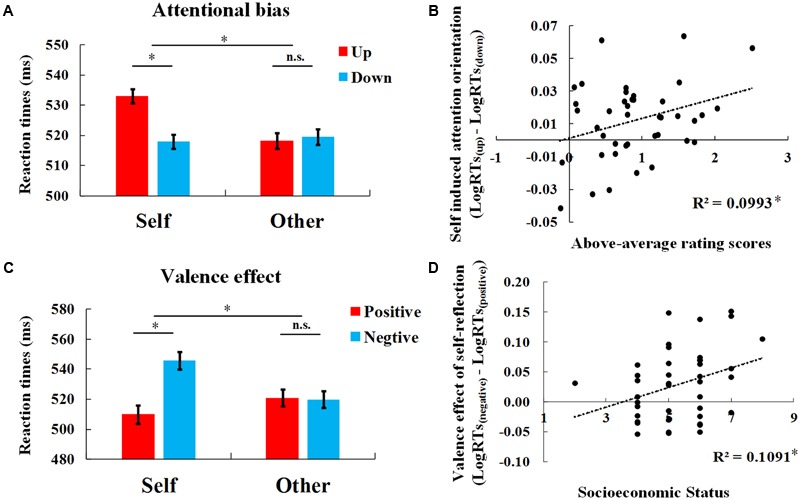
Results of Study 2. **(A)** RTs to targets at up and down positions after reflection. The RTs are reported in raw millisecond values to facilitate comprehension. Error bars represent within-subject standard error of the mean. **(B)** Correlation between downward attention orientation effects (lgRT_up_-lgRT_down_) after self-reflection and above-average scores. **(C)** RTs to targets after positive and negation reflection. The RTs are reported in raw millisecond values to facilitate comprehension. Error bars represent within-subject standard error of the mean. **(D)** Correlation between valence effect of self-reflection (lgRT_negative_-lgRT_positive_) and subjective SES. ^∗^*p* < 0.05.

The mean RTs and SDs are reported in **Table 1**, and the log-transformed RTs were subjected to a 2 (Person: self vs. disliked) × 2 (Valence: positive vs. negative) × 2 (Position: higher vs. lower) repeated measures ANOVA. The main effect of Valence was significant [*F*(1,40) = 5.208, *p* = 0.028, η^2^ = 0.115], with faster responses to the target after positive adjectives. The main effect of Person was not significant [*F*(1,40) = 0.608, *p* = 0.440, η^2^ = 0.015]. The main effect of Position was marginally significant with the tendency of faster responses to lower targets than higher targets [*F*(1,40) = 3.854, *p* = 0.057, η^2^ = 0.088]. The Person × Position interaction was significant [*F*(1,40) = 5.155, *p* = 0.029, η^2^ = 0.114]. A simple effect analysis showed that, after self-reflection, the RTs to lower targets were faster than to higher targets (*t*(40) = -3.318, *p* = 0.002, *d* = 0.520, 95% CI = [-.0194, -0.0047]), while for the disliked other, there was no difference (*t*(40) = 0.028, *p* = 0.978, *d* = .004, 95% CI = [-0.0078, 0.0080]; **Figure 3A**). The differential RTs to targets at up vs. down positions for individual subjects are demonstrated in **Supplementary Figure S1**. These results suggest a downward attention orientation after self-reflection, but not after disliked other-reflection. In addition, we also found a significant interaction between Person and Valence [*F*(1,40) = 5.253, *p* = 0.027, η^2^ = 0.116]. A simple effect analysis showed that, in the self condition, RTs to targets after positive adjectives were faster than after negative adjectives (*t*(40) = -3.178, *p* = 0.003, *d* = 0.496, 95% CI = [0.0107, 0.0480]), while this difference in the disliked other condition was not significant (*t*(40) = -233, *p* = 0.817, *d* = 0.037, 95% CI = [-0.0158, 0.0199]; **Figure 3C**). This interaction suggests a positive bias of self-reflection on the discrimination task. However, neither the interaction between Valence and Position [*F*(1,40) = 0.129, *p* = 0.721, η^2^ = 0.003], nor the three-way interaction of Person, Valence, and Position [*F*(1,40) = 0.016, *p* = 0.901, η^2^< 0.001] was significant.

Identical analyses were conducted on accuracy data. The 2 × 2 × 2 ANOVA showed significant main effect of Position [*F*(1,40) = 7.669, *p* = 0.008, η^2^ = 0.161] with higher accuracy for higher position than lower position. However, other main effects or interactions were not significant (*p*s > 0.1).

#### Above-Average Measurement

Participants showed a significant above-average effect, which was consistent with previous findings (Lee et al., 2010). Subjects thought they were better than the average level of the students at their university on the personality traits presented (*t*(40) = 9.495, *p* < 0.001, *d* = 1.483, 95% CI = [0.7036, 1.084]).

#### Correlations

Moreover, the downward attention orientation effect (lgRT_up_-lgRT_down_) after self-reflection were positively correlated with the above-average scores [*r*(41) = 0.315, *p* = 0.045; **Figure 3B**]. In addition, we also calculated the positive bias of self-reflection, using RTs to targets after presentation of negative adjectives minus those recorded after positive adjectives (lgRT_negative_-lgRT_positive_) in the self condition, and found that the positive bias of self-reflection was positively correlated with SES of the individual [*r*(41) = 0.330, *p* = 0.035; **Figure 3B**]. These correlation results suggest that the downward attention orientation after self-reflection linked with above-average self-view on personality traits. While the SES of the self was sensitive to the valence of the reflection.

Although the interaction between Valence and Position was not significant, the valence of the reflection modulated the association between the interdependence of individuals and the attentional bias. Interdependence was defined by the difference between the sum score of the 12 interdependent items and the sum score of the 12 independent items. Higher difference scores indicated greater interdependent cultural orientation (Ma et al., 2014). After positive self-reflection, individuals with higher interdependence showed stronger downward attention orientation effects [*r*(41) = 0.423, *p* = 0.006], while after negative self-reflection, the correlation was not significant and showed a negative tendency [*r*(41) = -0.199, *p* = 0.213]. Fisher-z transformation confirmed the correlations were significantly different (*z* = 2.85, *p* = 0.004).

### Discussion

The results of self-reflection in Study 2 replicated the results observed in Study 1, specifically that thinking about the self showed downward attention bias to targets appeared at a lower position. Through using a disliked other as a control, we clarified that the downward attention orientation effect specifically occurred after self-reflection, but not after disliked other-reflection. These results exclude the possibility that the downward attention orientation is general attention bias to targets appeared at lower position, or attention orientation after trait evaluation on all persons. Moreover, we found that individuals who regarded themselves as above average also showed stronger attentional bias after self-reflection, suggesting that the mechanism of the downward attention orientation after self-reflection is the psychological status within the social environment. These results were consistent with our hypothesis that thinking about the self would direct visual attention downward, and this effect was associated with a positive self-view.

Apart from the attention bias, we also found a positive bias of self-reflection. That is, positive self-evaluation resulted in faster responses than negative self-evaluation in the following target discrimination task, regardless of the targets’ position. Meier et al. (2004) showed an association between brightness and evaluation of objects (i.e., bright objects are good, whereas dark objects are bad). Consistent evaluations (e.g., good–white) speeded responses, while inconsistent evaluations (e.g., bad–white) delayed responses, suggesting that evaluations could activate perceptual cues (i.e., brightness variations). People have an automatic association between the self and positivity, known as the self-enhancement or self-serving bias (Alicke, 1985; Taylor and Brown, 1988; Chambers and Windschitl, 2004). Thus, it was reasonable that an inconsistent description (e.g., self-negative) induced conflict, which interfered the discrimination task. While the consistent pairs (e.g., self-positive) required less mental resources, making it easier to respond to targets. For others, there are no specific associations of other-positive or other-negative. Therefore, no valence effect was found after other-reflection. Moreover, it was found that the positive bias of self-reflection was stronger for individuals with higher SES. This correlation suggests that individuals with higher SES possess a stronger positive association of the self than individuals with lower SES. This finding is consistent with the results that individuals with higher SES, especially young adults, reported higher self-esteem (Twenge and Campbell, 2002). However, the valence effect in Study 1 was not significant (*p*s > 0.1), but displayed similar patterns for both self and friend (i.e., faster responses to targets after positive traits than negative traits, **Figure 2C**). One possible reason for the increased valence effect in Study 2 compared to Study 1 was the presence of the disliked other. A disliked other may have served as a potential threat that increased the motivation to defend from negative self-evaluation. When participants were only evaluating themselves and/or their friend, the salience of valence information may have decreased, resulting in the null valence effect observed in Study 1.

In addition, the downward attention orientation effects after self-reflection of positive traits were stronger for individuals with higher interdependence. However, this association tended to be reversed after self-reflection of negative traits. For the role of the interdependent self-construal, there is evidence that the effect of social influences on the self-concept are stronger in individuals with interdependent self-construals rather than independent self-construals (Liew et al., 2011). Thus, when thinking about self, especially when considering positive traits, individuals with higher interdependence were more likely to activate a high social status of the self relative to others, which resulted in stronger attentional bias. However, for negative self-reflection, participants were more likely to perceive the negative self-description as a kind of self-threat for interdependent individuals (Park and Kitayama, 2014), under which the positive self-view was reduced, resulting in a weaker attentional bias.

## General Discussion

Our results in Studies 1 and 2 demonstrated that self-reflection directed visuospatial attention downward, which were consistent with our hypothesis. In Study 1, similar attention orientation effects were observed in both friend- and self-reflection. While in Study 2, reflection on a disliked other showed a null effect of attentional orientation, which ruled out the possibility that the downward attention orientation after self-reflection was a general facilitation to lower targets. Of note, the attention orientation effects observed after self-reflection were stronger for individuals that thought of themselves, to a large extent, above average.

The consistent effects of self-reflection in Studies 1 and 2 suggest that ones’ self-concept contains vertical spatial information. It might be that the “above-average” self-schema associated self-concept with a higher status relative to others, as a type of self-enhancement to maintain self-esteem (Heine et al., 1999). In a previous work, a new learned association of the self and a perceptual symbol (a geometric shape) enhanced sensitivity to the self-relevant stimuli in a perceptual matching task (Sui et al., 2012). In our study, the association of self-concept and high status is deep-seated and learned over long periods of time. Thus, according to the PSS theory (Barsalou, 1999), it was reasonable that reminding of the self activated the higher social status, which is a spatial symbol of the self. With others below the self as the frame of reference, the high position one possesses resulted in downward attention orientation to see others below the self, which would likely strengthen the sense of pride. In addition, the null results of the disliked other clarified that the downward attention orientation effect was not a general facilitation effect to targets appearing at the lower position. Self-reference effect demonstrated self-reflection could affect memory (Rogers et al., 1977; Klein and Kihlstrom, 1986). Our results provide first evidence that the effect of self-reflection on personality could extend to attention, which enriches our understanding of the superiority of self.

However, the attention effects of other-reflection in Studies 1 and 2 were different. The downward attention orientation occurred after friend-reflection (Study 1), but not after disliked other-reflection (Study 2). One conceptual difference between a friend and a disliked other is that a friend may be considered part of the self for individuals with an interdependent self-construal, while the disliked other is decidedly considered a separate entity (Markus and Kitayama, 1991). Since the results of self-reflection suggested an association between downward orientation of attention and self-enhancement, and since this self-enhancement could extend to close others construed as part of the self (Gardner et al., 2002), it was not surprising that reflecting upon a close other (i.e., friend) showed similar effects as self-reflection. We noticed that our participants were more interdependent than independent, and the more that participants liked their friend, the stronger the attention orientation effect that they exhibited. These findings support the inference that the friend-reflection directed visual attention downward because the friend was regarded as part of the self. Our participants were all from an Eastern culture (i.e., China). It is possible that participants from a Western culture, who do not necessarily consider friends as part of self, may not exhibit the same downward attention orientation effect after friend-reflection.

Taking the cultural orientation or self-construal into account, we speculate the self-related attention orientation would show some culture differences. As is stated above, the attention effect of the self depends on two factors: ones’ positive self-view and the sensitivity to social context. The downward attention orientation will occur only when the positive self-view (e.g., I am clever) was held in social comparison (e.g., I’m cleverer than others). It is well known that the positive self-view and/or self-enhancement is more typical in Western cultures than Eastern cultures (Gardner et al., 2002). However, there has also been some evidence that individuals in Western culture are less sensitive to social influences, i.e., they are less likely to compare themselves with others to define the self (Markus and Kitayama, 1991; Heine et al., 1999; Liew et al., 2011). Thus, the culture difference of the self-related attention orientation depends on which factor, i.e., positive self-view or sensitivity to social context, dominants the effects. Future research is needed to investigate this issue in more depth.

Although the downward attention orientation effects after self-reflection were found consistently in Studies 1 and 2, we noticed that the differential RTs to up and down targets were quite small (9–15 ms). The small differences of RTs were also shown in previous work using the target discrimination paradigm. For example, in Chasteen et al. (2010), God/Devil facilitated target discrimination in up/down (vs. down/up) position. The differential RT (up minus down) was 14 ms in God condition and -5 ms in Devil condition. There were also some studies showed relatively larger differential RTs. For example, Estes et al. (2008) showed head/foot related words facilitated target discrimination in up/down position (vs. down/up position). The size of the difference was about 40 ms. According to the PSS theory (Barsalou, 1999), the attention orientation effect depends on the association of the concept and its spatial information. Head/foot are concrete concepts explicitly associated with up/down positions, while god/devil and self/other are abstract concepts only implicitly associated with up/down metaphors. Since the association of self-concept and higher position one possesses is not quite tight, i.e., depends one cultural orientation and social context, it was not surprising that the attention orientation effect after self-reflection was small, and was not found for all subjects.

According to previous findings, one thing that should be addressed is whether the attention orientation effect observed was manifestations of facilitation (Meier and Robinson, 2004; Chasteen et al., 2010; Zanolie et al., 2012) or interference (Bergen et al., 2007; Estes et al., 2008). The two types of RTs to targets at the compatible locations were shorter or longer, than at the incompatible location. If the effects can be conceptualized as interference, and not facilitation, the faster responses to targets at the lower position after self-reflection might indicate an upward attention orientation. Gozli et al. (2013) clarified that interference would not occur unless the procedure met two conditions. The first is the use of multiple concept categories (e.g., clothing, house, animals). Multiple categories might lead to a between-trial category switching cost, and might prevent stimulus-response mappings between a subset of the category and a target location. The second is the use of short cue–target stimulus onset asynchrony (SOA; 150–350 ms). In the current study, we used only trait adjectives as a single concept category, and the self and other conditions were in different blocks, with a 10 s interval to prevent unwanted category switching. We also used a long SOA, with the time from the cue word reminding self or others to the target appearing being 1050 ms. Therefore, we believe that the results observed were facilitation effects, not interference. That is, the faster responses to lower targets after self-reflection suggest a downward attention orientation, which was consistent with our hypothesis.

One may argue that the valence of the person might drive the attention effects, i.e., reflection on a positive person (self and friend) induced downward attention orientation effect, while reflection on a negative person (disliked other) showed null effect. The affective valence of a person is individuals’ subjective attitudes to the self and others. Actually, the positive attitude to the self is just what we called “positive self-view” or self-enhancement, which is the theoretical bases for the attention effect we found. One limitation of current study is we did not include a neural person as control. The friend could be regarded as part of self and share the positive association with the self, while the disliked other is obviously negative to the self. Therefore, whether a neutral person could induce downward attention orientation needs to be tested in future research. Another direction of future research is investigating how to induce the attention orientation by priming the positive or negative view of self or others. Resent work of “good true self” proposes that people tend to believe that every person (both self and others) is motivated to behave in morally good ways (Newman et al., 2015; De Freitas et al., 2017a). This positive belief ties to moral essence of a person and is consistent across cultures (De Freitas et al., 2017b). In future research, we could use morally good events to induce the positive belief about others, and test whether the belief of “good true self” could result in attention orientation.

## Conclusion

Our study demonstrated the visuospatial influence of self-reflection, provided the first evidence that thinking about the self directs attention in a downward fashion, and that this attentional bias was associated with an above-average self-evaluation.

## Ethics Statement

This study was carried out in accordance with the recommendations of Shenzhen University with written informed consent from all subjects. The protocol was approved by the ethics committee of Shenzhen University.

## Author Contributions

YL collected and analyzed data, wrote the paper. YT provided theoritical bases of the study and revised the manuscript. HL paid for the study and revised the manuscript.

## Conflict of Interest Statement

The authors declare that the research was conducted in the absence of any commercial or financial relationships that could be construed as a potential conflict of interest.

## Supplementary Material

FIGURE S1 Attention orientation effect of self- and other-reflection for each participant in Study 1 and Study 2.
