# Supplementary figures and images for: Self-reflection Orients Visual Attention Downward
Source: Front Psychol. 2017 Sep 5;8:1506. doi: 10.3389/fpsyg.2017.01506 (PMC5591849; doi:10.3389/fpsyg.2017.01506)

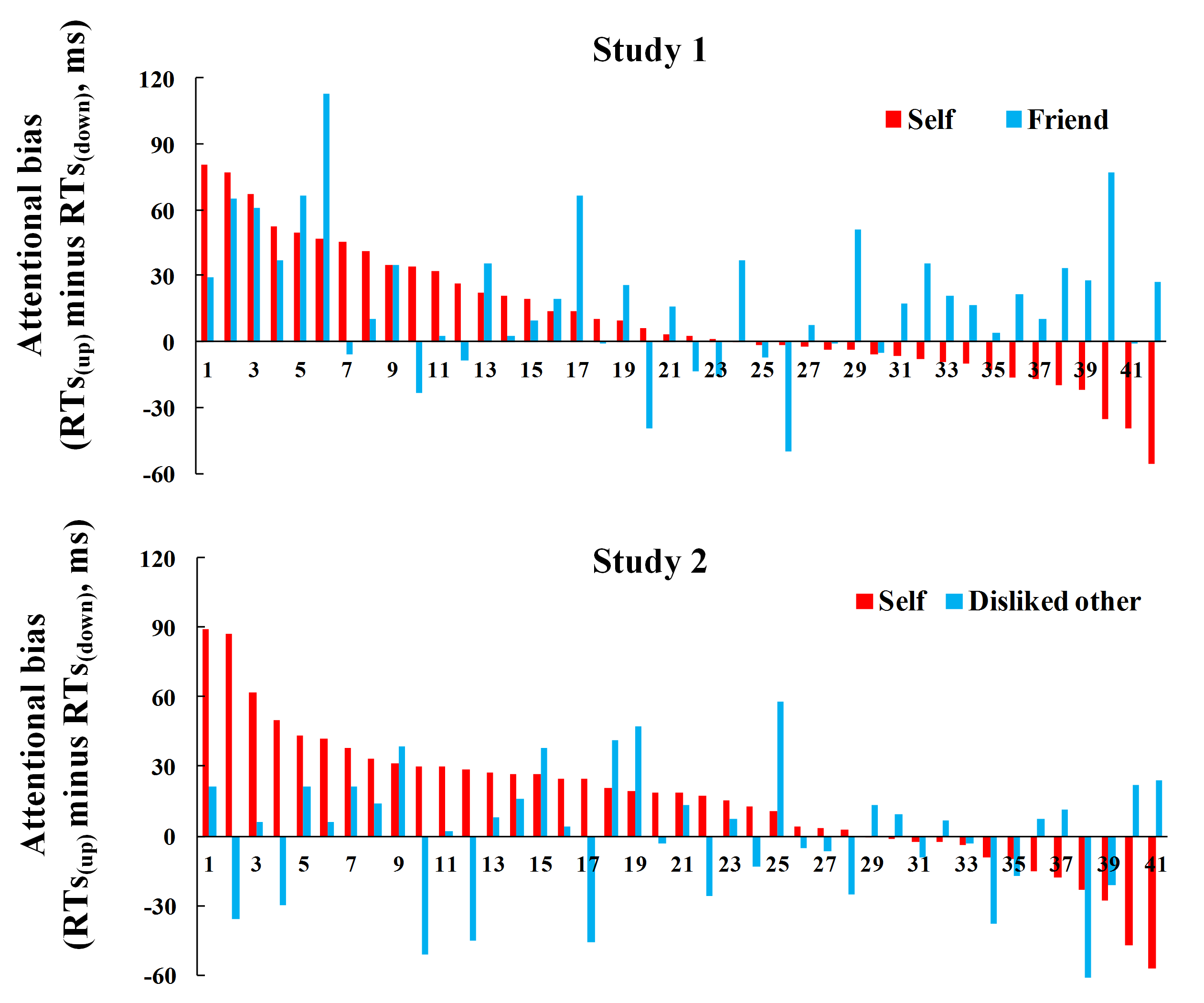

Supplement: FIGURE S1 — Attention orientation effect of self- and other-reflection for each participant in Study 1 and Study 2. [file Image_1.TIF]
